# Is Iron Supplementation Influenced by Sub-Clinical Inflammation?: A Randomized Controlled Trial Among Adolescent Schoolgirls in Myanmar

**DOI:** 10.3390/nu11040918

**Published:** 2019-04-24

**Authors:** Min Kyaw Htet, Umi Fahmida, Drupadi Dillon, Arwin Akib, Budi Utomo, David I Thurnham

**Affiliations:** 1 South East Asian Ministers of Education Organization, Regional Center for Food and Nutrition (SEAMEO RECFON) Pusat Kajian Gizi Regional, Universitas Indonesia, Jakarta 10430, Indonesia; umifahmida@gmail.com or ufahmida@seameo-recfon.org (U.F.); ddrupadi@hotmail.com (D.D.); 2 Township Health Department, Ministry of Health Myanmar, Hakha 03011, Chin State, Myanmar; 3 Sydney School of Public Health, Sydney Medical School, The University of Sydney, Sydney NSW 2006, Australia; 4 Faculty of Medicine, University of Indonesia, Jakarta 10430, Indonesia; aralpur@yahoo.com; 5 Faculty of Public Health, University of Indonesia, Depok 16424, Indonesia; budi.utomo.ui@gmail.com; 6 Northern Ireland Centre for Food and Health, School of Biomedical Science, University of Ulster, Coleraine BT52 1SA, UK; di.thurnham@ulster.ac.uk

**Keywords:** anemia, iron deficiency, Myanmar, sub-clinical inflammation

## Abstract

Iron absorption was impaired in the presence of sub-clinical inflammation (SCI) and might hamper the effect of iron supplementation. The purpose of the study was to identify the influence of SCI on iron supplementation. A randomized, double-blinded, placebo-controlled experimental study was conducted among anaemic adolescent schoolgirls in Ayeyarwady region, Myanmar. A total of 402 schoolgirls were recruited from six schools screened from 1269 girls who were assigned into one of four groups: Folate group (2.5 mg of folate), Vitamin A group (15,000 IU of vitamin), Iron folate group (60 mg elemental iron and folate) and Iron, and vitamin A and folate group. Supplementation was done once a week for 12 weeks. Iron, vitamin A and inflammation were measured at the baseline, middle and endline. Changes in serum ferritin and body iron were significantly higher in the IFA and IFA + vitA among those without SCI. There was interaction between vitamin A and SCI on Hb changes. Analysis of GLM repeated measure showed interactions between treatment and SCI for hemoglobin and serum transferrin receptor. Those treated with vitamin A had better outcomes when there was SCI. Inflammation accompanied a negative effect on iron supplementation and vitamin A improved efficacy of iron supplementation in the presence of SCI.

## 1. Introduction 

Despite the progress in anemia control over the last decades, iron deficiency and anemia still remain major nutritional problem in many parts of the world [1]. Fortification and food based approaches are promising strategies to overcome the problem but iron supplementation remains important since it is the most effective strategy for the areas where iron deficiency is common and anemia is a major public health problem [2,3]. Anemia and iron deficiency are prevalent in developing countries and are often accompanied by infections and sub-clinical infection (SCI). The interaction between infections and nutritional status of individuals is well recognized and in recent years the relationship between the host iron status and infections has become a focus of research interest [4]. Not all studies have shown iron supplementation to be effective in reducing anemia and it may even cause deleterious health outcomes in the area where infections, particularly malaria, are common as, for example, in the Pemba trial [5]. Subsequently to that study, WHO in 2006, recommended targeted and supervised supplementation in malaria endemic areas only to iron deficient or severely anemic young children [6]. However, in the low resource settings where anemia and iron deficiency are prevalent, it is not always feasible to assess the iron status of the population before giving supplementation. 

Understanding of the interaction between infections and iron status is gradually improving yet the work remains challenging. The function of the newly-discovered, iron-regulatory hormone hepcidin explains why anemia commonly occurs in the presence of chronic diseases and infections [7]. While the debate is ongoing for iron supplementation in malaria-endemic areas, little attention is paid to the importance of sub-clinical inflammation (SCI) in relation to the effectiveness of iron supplementation. SCI can exist in the apparently-healthy population and unless taken into account, its influence on anemia will be overlooked [8]. Studies showed that SCI can interfere with the assessment of micronutrient status especially indicators of iron and vitamin A status [9,10,11]. However, there have been no attempts to investigate the influence of SCI on the effectiveness of iron supplementation. Studies have shown additional benefits in reducing anemia by combining iron and vitamin A, but none have considered the role of SCI or anti-inflammatory properties of vitamin A on the mechanisms involved.

We hypothesized that the effect of iron supplementation on anemia may not be optimal in the presence of SCI and explored the interactive effect of combined iron and vitamin A supplementation and SCI. The study was registered at the clinical trial registration system with the identifier ClinicalTrials.gov ID: NCT 01198574.

## 2. Materials and Methods

Study setting and population: Background information on the subjects and study area are described elsewhere [12]. The study was conducted in Nyaung Done Townhsip, a peri-urban area in Ayeyarwady division, the delta region of Myanmar located about 3 h drive, (~40 km) distance from the former capital city Yangon.

The subjects were 8 to 10 grade post-menarcheal, adolescent school girls and were recruited in July 2010. Screening for anemia, malaria and haemoglobinopathies was done among the schoolgirls (*n* = 1269) from six schools in the study area during the recruitment (5). Postmenarcheal schoolgirls with anemia (Hb < 12.0 g/dL), not suffering from any major illness or known disease at the time of recruitment, were invited to join the study. Those with severe anemia (Hb < 7.0 g/dL) or those who took multivitamin supplements regularly for past three months were not eligible (2). The girls with severe anemia were treated with iron tablets and not included in the study. Deworming with a single dose of albendazole (400 mg) was done in these girls one week before the blood collection.

Screening for anemia, hemolytic anemia and malaria infection was done in 6 schools among 8 to 10 grade schoolgirls and details were reported elsewhere [13]. The sample size was calculated in order to detect a mean hemoglobin difference of 5 g/L between two groups, with 95% power at 5% level of significance based on a previous study [14]. The sample size was 73 for each group and allowed for a dropout rate of 20%. To take account of inflammation, the sample was increased to 100 and a total of 402 subjects for four interventions were recruited. Deworming with a single dose of Albendazole (400 mg) was done to these girls one week before the blood collection.

Study design and Intervention: The study was a randomized, double blinded, placebo controlled trial and the subjects in all 6 schools were randomly assigned into one of the four treatment groups using random allocation software with the block size of 8. The block randomization was done by computer generated randomization and was done by a statistician. The randomization list was kept by someone who was not involved in the study. Allocation concealment was done by enclosing the assignment of treatment in sequentially numbered, opaque, sealed envelopes. This allocation concealment was done to prevent selection bias and it was done at the time of enrolment to protect the assignment sequences. The study personnel and participants were blinded to treatment assignment for the duration of the study.

There were four treatment groups involved in the study and each group received two tablets as follows: IFA group (60 mg iron with 2.5 mg folate and vitamin A placebo), VA group (VA 15,000 IU and placebo iron i.e., 2.5 mg folate), IFA+VA group (60 mg iron with 2.5 mg folate and VA 15,000 IU), and placebo group (FA) (2.5 mg folate and vitamin A placebo). The intervention groups were equivalent to 2 by 2 design with iron and vitamin A, but with all the groups receiving folate to ensure that anemia due to folate deficiency was controlled for. Supplementation was given once a week for 12 weeks and the weekly dosage of iron folate corresponded to previous studies [15] and that of vitamin A was calculated based on RDA (Recommended Daily Allowance). Supplements were prepared for the study by Kimia Farma Pharmaceutical Factory, Indonesia and placebos were made identical in appearance to the supplements and were coded at the factory. All the personnel and investigators were blinded and de-blinding of the code was done only after the preliminary data analyses were done.

Compliance: Supplementation was done once a week for twelve weeks under close supervision of school teachers. During the intervention, principal investigator and field assistants visited the schools every week to monitor any side effects, to check the compliance, and to collect empty supplement containers and provide the supplements for the upcoming week.

Data Collection: At baseline, participants were administered a standard interview using structured questionnaire. Anthropometric assessments were carried out by two trained persons. Body weight was measured by flat electronic weighing scale (SECA 874, Hamburg, Germany) to the nearest 0.1 kg and height measured by measuring tape (Microtoise SECA 206, Hamburg, Germany) to the nearest 0.1 cm. Anthropometric nutritional status was assessed using z scores and WHO anthroplus software was used for the analysis [16]. Venous blood samples were collected three times at baseline, midline, and endline (week 0, week 6 and after week 12) of the study. In the presence of a physician, phlebotomy was done by experienced nurses and 3 mL of non fasting venous blood was taken from each subject in the morning school session 8:00 to 12:00 hr. The blood samples were collected into a non-heparinised vacuette as well as into an EDTA tube and transported in ice-cooled containers to the laboratory of Department of Medical Research, Lower Myanmar (DMR-LM) within 4 h.

### 2.1. Ethics and Registration

Ethical approval of the study was granted from Faculty of Medicine, University of Indonesia (128/PT 02.FK/ETIK/2010) and also from Department of Medical Research, Lower Myanmar (18/Ethics 2010 DMR-Lower Myanmar). The study was registered at the clinical trial registration system at ClinicalTrials.gov (identifier ID: NCT 01198574).

Laboratory analysis: Hemoglobin was measured by cyanmethemoglobin method at Nutrition Research Division, DMR-LM. The quality control for the Hb assessment was done with control samples of known values from National Health Laboratory on daily basis and the coefficient of variation of the assessment was below 5.0%. Blood samples were centrifuged at 1500× *g* for 10 min and serum samples were kept in polyethylene vials at –20C before further analyses were done. These samples were kept frozen in dry ice during the shipment to the laboratory of South East Asian Ministers of Education Organization-Regional Center for Food and Nutrition (SEAMEO-RECFON), University of Indonesia, Jakarta, Indonesia for further analyses. Details of these analyses were described in a previous paper [12]. Briefly, an in-house sandwich enzyme-linked immunosorbent assay was used to measure serum ferritin (SF), transferrin receptor (sTfR), retinol binding protein (RBP), C-reactive protein (CRP), and α1-acid glycoprotein (AGP) on 200 µL serum [17]. Body iron store was calculated using the method of Cook et al. [18]. Serum retinol concentrations were measured by high performance liquid chromatography [19]. Serum folate concentrations were measured using microbiological assays according to the methods of O’Broin and Kelleher [20].

Anemia was defined as haemoglobin <12.0 g/dL [21], iron deficiency (ID) when SF < 15 µg/L and /or sTfR was >8.5 mg/L and IDA by the occurrence of ID and anemia [11,22]. Low vitamin A status was defined when serum retinol concentrations were <1.05 µmol/L) [23]. SCI was defined when serum CRP>5mg/L and/or AGP concentrations were >1g/L at any time throughout the study (baseline, midline, endline) [9,10].

### 2.2. Statistical Analysis

Statistical analyses were performed using statistical software package SPSS (SPSS; Chicago, IL USA) Version 15.0 for windows. Normality of distribution of the variables was checked with Komogorov-Smirnov test and serum ferritin, sTfR, and serum retinol were log transformed to better approximate a normal distribution. Data were presented as means ± SD or geometric means ± SD.

The baseline biochemical status of subjects among 4 groups was compared by one-way analysis of variance (ANOVA). Posthoc analysis using LSD was applied when ANOVA was statistically significant. The prevalence of iron deficiency, low vitamin A status and SCI among the groups was compared using Chi-square test. The change in Hb, serum ferritin and body iron store were compared among the four treatment groups using ANOVA test after stratification by SCI. Multiple linear regression was performed to identify the determinants of changes in Hb. In the regression model, baseline concentration of Hb, serum ferritin and transferrin receptor were controlled and treatment effect of iron, vitamin A and their interactions with SCI were included. A general linear model (GLM) with repeated measurements was done to investigate the effect of intervention on the outcome variables such as Hb, iron and vitamin A status and the interaction between the treatment groups and the SCI. The outcome variables were treated as within subject variables. The type of treatment and SCI were treated as between subject variables. The analysis was done with and without including the interaction of treatment and inflammation (treatment × inflammation).

## 3. Results

Of the 402 recruited subjects, 392 completed the 12 week intervention study but complete data were only available for 391 subjects which was used for the analyses. The main reason for the drop-outs was due to resignation from the schools and data for lost subjects were not different from the remaining subjects. The girls were closely monitored by teachers and the compliance was high (86.5%) with no difference between group. The detail description of baseline characteristics of subjects were described elsewhere [12]. The anthropometric and biochemical status of the subjects were compared among the four groups and there were no significant differences (Table 1).

Change in Hb, serum ferritin and body iron store were compared among the four groups after stratification by SCI (Table 2). The results showed no significant difference in Hb change between the 4 treatments although there was a tendency of greater increase in Hb in IFA and IFA + vitA group (p value=0.139) in subjects without SCI, but not in the group with SCI. Regarding the change in serum ferritin and body iron store, there were significant differences between treatment groups in subjects without inflammation and the changes were higher in the IFA and IFA+ VitA and folate groups. However, there were no significant differences between treatments for the changes in Hb, ferritin, or body iron stores in subjects with SCI.

Table 3 shows the results of regression analysis for the outcome Hb change in response to iron or vitamin A alone or interaction with inflammation. The result shows treatment with iron significantly increased Hb concentration (*p* = 0.002), but not in the presence of inflammation (*p* = 0.268). Vitamin A alone in those with no inflammation did not increase Hb, but there was a significant increase in Hb in those subjects with inflammation.

The results of analysis by general linear model with repeated measures for subjects with and without inflammation are shown in Table 4. There were significant differences in the increases in Hb (*p* < 0.027) and sTfR (*p* < 0.013) concentrations in response to the different treatments between those subjects with and without inflammation. The results showed that in the group with SCI, subjects who received either vitamin A alone or together with IFA had better iron status compared to the other groups.

## 4. Discussion

The important finding of the study is that the effect of iron supplementation can be hampered by SCI and the addition of vitamin A can be an advantage in that condition.

The study was conducted in the delta region Myanmar where the prevalence of anemia was reportedly high [24]. Indeed, the finding from the screening phase showed the prevalence of anemia was ~ 60% in the area which confirmed anemia as a major public health problem in the area [13]. Myanmar is a country situated in South East Asia with the population of approximately 51 millions according to census 2014 [25]. About 70% of the populations reside in rural area and majority of them are working in the agricultural sector. The results showed that around 30% of the subjects were iron deficient (serum ferritin <15 µg/L and/or sTfR >8.5 mg.L) and had low vitamin A status (serum retinol <1.05 µmol/L) (Table 1). The prevalence of thinness (BMI-for-age < e-2) was 8.9% among the girls which was lower than findings from recently conducted nationwide micronutrient survey (18% prevalence of thinness for union level) [26].The subjects from the study area were relatively healthy and as a result, the prevalence of inflammation was as low as 6%. This was in contrast with our previous work from Indonesia where the prevalence of inflammation was almost 40% among high school girls of the same age group [27].

However, despite the low prevalence, the finding showed the significant negative impact of inflammation on effectiveness of iron supplementation. Table 2 shows the influence of SCI on the effect of iron supplementation for the changes in Hb, serum ferritin and body iron at the end of the 12 week supplementation. The finding suggested that effect of iron supplementation on serum ferritin and body iron store were impaired by SCI. Inflammation is our body physiological response to infections and injuries and the purpose is to minimize the damage to our body and to facilitate the repair process [28]. Unlike in clinical settings, sub-clinical inflammation (SCI) can be always present among the apparently healthy population and unless taken into account, it will lead to misinterpretation of the biomarkers of nutritional status assessment [9,10]. Few studies have investigated the association between sub-clinical inflammation and anemia, but were mostly inconclusive and descriptive. A recently published paper showed subclinical inflammation (AGP>1.0 g/L) was a risk factor for anemia among the Cambodian children and the author suggested that there might be inhibition of iron absorption as well as the release of stored iron as a result of inflammation [29]. Another study from Papua New Guinea showed the children who had high CRP (>5 mg/L) and AGP (>1.2 mg/L) had high prevalence of anemia [30]. While it failed to reach statistical significance (*p* = 0.053), a study from Nicaragua showed the risk of anemia was 1.5 times higher for the subjects with high acute phase protein (AGP > 1.0 g/L) than the normal subjects [31]. Similarly, the finding from our study indicates that SCI is an important determinant that undermines the effectiveness of iron supplementation.

The mechanism by which inflammation interferes with the iron supplementation might be explained by the negative iron regulatory hormone hepdicin, which inhibits intestinal iron absorption, release of storage iron from the reticuloendothelial system. During inflammation, hepcidin is released mainly from the liver with the stimulation from pro-inflammatory cytokines especially IL-6 [7,32]. It is also suggested that proinflmammatory cytokines suppress the production or biological activity of erythropoietin in anemia of inflammation [33]. However, studies have not been done to investigate the influence of SCI on iron supplementation and despite the low prevalence of SCI, we have shown that SCI interfered with the effectiveness of iron supplementation.

Vitamin A has been extensively studied for its important roles in body’s physiological and immune functions yet still many issues remain unsolved and the data is limited especially for this age group. The combined effect of vitamin A and iron supplementation was well documented in previous studies and studies showed that vitamin A is essential for the mobilization of storage iron and a possible role in enhancing the erythropoietin [34,35]. The regression analysis for the outcome Hb change shows the interaction effect between vitamin A and SCI (vitamin A × SCI) (Table 3). Although we cannot conclude with certainty, the possible mechanism might be the immunomodulatory function of vitamin A whereas it enhances Th2 anti-inflammatory pathway while minimizing the Th1 pro-inflammatory pathway [36]. Moreover, this effect may be an adjuvant function of vitamin A on iron supplementation.

Table 4 shows the effect of iron supplementation is hampered by SCI. The important finding is the significant interaction effect between the treatment and SCI (treatment × SCI) for the outcome hemoglobin and serum transferrin receptor. Specifically, when there was SCI, those who received vitamin A such as vitamin A group and vitamin A + IFA group showed significantly better outcome for Hb and sTfR compared to the other groups. In this study, both hemoglobin and serum transferrin receptor concentration were not influenced by SCI but when accompanied by SCI, vitamin A containing group showed better responses for these indicators. There was no interaction between iron and SCI. On the other hand, when there was no SCI, IFA, and vitamin A +IFA group showed better outcomes. These findings suggested that iron supplementation would be more effective regardless of vitamin A if there is no inflammation. However, once there is inflammation, the additional vitamin A supplementation is an advantage.

The significance of the study is that the effect of iron supplementation on iron status can be observed in the two scenarios stratified by the inflammation status. The prevalence of SCI was relatively low in this study, but there might be seasonal increases that should be further explored. The results of this study indicate that SCI has programmatic implications for the effectiveness of iron supplementation where infections are common. Given SCI is not commonly assessed in programmatic setting, additional vitamin A may be an advantage to improve the effectiveness of IFA supplementation.

## Figures and Tables

**Table 1:** Anthropometric and biochemical status of the subjects at baseline of study ^1^.

	Treatment Group					
Variables	FA (*n* = 98)	VitA (*n* = 101)	IFA (*n* = 94)	IFA + VitA (*n* = 98)	Total *n* = 391	*p* ^3^
Age (years)	15. 8 ± 1.13	16.1 ± 1.20	15.9 ± 1.21	16.0 ± 1.15	15.9 ± 1.17	0.270
Age of Menarche	13.2 ± 0.89	13.3 ± 0.89	13.1 ± 1.01	13.1 ± 0.89	13.2 ± 0.92	0.469
Weight (kg)	41.3 ± 6.12	42.3 ± 6.42	41.5 ± 6.03	41. 8 ± 6.27	41.7 ± 6.20	0.711
Height (cm)	149.8 ± 4.60	151.7 ± 5.75	150.8 ± 5.33	151.2 ± 5.23	150.8 ± 5.27	0.075
BMI-for-Age	−0.67 ± 0.96	−0.73 ± 0.86	−0.77 ± 0.94	−0.79 ± 1.02	−0.74 ± 0.94	0.821
Thinness (%) ^4^	10.2	5.9	9.6	10.2	8.9	0.672
Hemoglobin (g/dL)	8.9 ± 1.3	8.9 ± 1.1	8.8 ± 1.1	8.8 ± 1.2	8.9 ± 1.2	0.850
Serum Ferritin ^2^ (µg/L)	29.9 ± 2.5	33.4 ± 2.5	31.4 ± 2.4	25.5 ± 3.1	29.9 ± 2.6	0.233
Transferrin receptor (mg/L)	6.74 ± 1.5	6.56 ± 1.42	7.09 ± 1.52	7.16 ± 1.59	6.9 ± 1.5	0.391
Body iron (mg/kg)	4.04 ± 3.77	4.49 ± 4.21	3.97 ± 3.91	3.16 ± 5.27	3.92 ± 4.34	0.185
Serum retinol ^2^ (µmol/L)	1.18 ± 1.28	1.17 ± 1.31	1.21 ± 1.28	1.18 ± 1.29	1.18 ± 1.29	0.858
Serum folate (nmol/L)	6.02 ± 1.95	6.0 ± 1.7	6.66 ± 1.80	6.27 ± 1.67	6.22 ± 1.78	0.574
CRP(mg/L)	0.73 ± 2.07	0.96 ± 3.32	1.38 ± 6.08	0.47 ± 0.87	0.88 ± 3.60	0.349
AGP (g/L)	0.76 ± 0.18	0.75 ± 0.16	0.74 ± 0.20	0.74 ± 0.17	0.75 ± 0.18	0.798

^1^ mean ± SD or geometric mean ± SD, FA: Folate group, VitA: Vitamin A and folate, IFA: Iron and folate group, IFA + vitA: Iron folate and vitamin A group; ^2^ Serum ferritin and serum retinol were adjusted for sub-clinical inflammation using the meta-analysis correction factor; ^3^ one-way ANOVA was used for between groups comparison, values are significantly different at *p* < 0.05(Bonferroni correction); ^4^ Thinness was defined by BMI-for-age z score <−2.

**Table 2:** Changes in Haemoglobin, serum ferritin and body iron store with or without sub-clinical inflammation at the end of 12 week iron supplementation ^1^.

	Change in Hb		Change in SF (Log Value)		Change in Body Iron Store	
	No SCI ^2^ (336)	SCI (55)	No SCI (336)	SCI (55)	No SCI (336)	SCI (55)
FA	1.98 ± 1.08	2.18 ± 1.14	0.91 ± 1.58 ^a^	1.05 ± 2.59	−0.24 ± 1.90 ^a^	−0.47 ± 1.56
VitA	1.96 ± 1.25	2.12 ± 1.66	1.04 ± 1.65 ^a^	0.89 ± 1.87	0.28 ± 1.92 ^a^	−0.10 ± 2.22
IFA	2.28 ± 1.18	2.10 ± 1.20	1.26 ± 1.54 ^b^	1.05 ± 2.59	1.20 ± 1.64 ^b^	0.43 ± 3.23
IFA + vitA	2.28 ± 1.32	2.46 ± 1.40	1.41 ± 1.72 ^b^	1.07 ± 1.99	1.73 ± 2.45 ^b^	0.78 ± 2.95
F value	1.843	0.186	13.55	0.303	16.477	0.688
*p* value ^3^	0.139	0.906	<0.001	0.823	<0.001	0.563

^1^ mean ± SD for Hb change and body iron store, geometric mean ± SD for serum ferritin change, ^ab^ Values with different superscript letters are significantly different, *p* < 0.05, FA: Folate group, VitA: Vitamin A and folate, IFA: Iron and folate group, IFA + vitA: Iron folate and vitamin A group; ^2^ SCI = sub-clinical inflammation defined by CRP > 5 mg/L or AGP > 1 g/L at any time throughout 12 week of intervention study; ^3^ one-way ANOVA was used for between groups comparison, values are significantly different at *p* < 0.05.

**Table 3:** Determinants for Hb changes from baseline to endline of 12 weeks Intervention.

	Unstandardized Coefficient (B)	SE	Standardized Coefficient	*p*
**Treatment with Fe ^1^**				
(Constant)	11.19	0.51		<0.001
Hb at baseline	−0.85	0.04	−0.81	<0.001
Serum ferritin at baseline	−0.09	0.11	−0.03	0.397
sTfR at BL	−1.85	0.27	−0.27	<0.001
Treatment with Iron	0.28	0.09	0.11	0.002
SCI treated with Fe	−0.26	0.24	−0.05	0.268
SCI	0.11	0.16	0.03	0.490
**Treatment with VitA ^2^**				
(Constant)	11.37	0.51		<0.001
Hb at baseline	−0.85	0.04	−0.82	<0.001
Serum ferritin at baseline	−0.11	0.11	−0.04	0.324
sTfR at BL	−1.77	0.27	−0.25	0.000
Treatment with VitA	−0.08	0.09	−0.03	0.359
SCI treated with VitA	0.48	0.24	0.10	0.044
SCI	−0.26	0.17	−0.07	0.123

^1^ Linear regression for the change in haemoglobin concentration by treatment with Iron, adjusted for baseline Hb, baseline serum ferritin and sTfR, R^2^ = 0.57, F(89.34), (*p* < 0.001); ^2^ Linear regression for the change in haemoglobin concentration by treatment with Vitamin A, adjusted for baseline Hb, baseline serum ferritin and sTfR, R^2^ = 0.57, F(86.97), (*p* < 0.001).

**Table 4:** Effect of iron and vitamin A supplementation in the presence and absence of sub-clinical inflammation among the adolescent girls during 12 weeks of intervention ^1^.

	Without Sub-Clinical Inflammation				With Sub-Clinical Inflammation				*p* ^2^			
Variables	FA (*n* = 82)	VitA (*n* = 86)	IFA (*n* = 82)	IFA + vitA (*n* = 86)	FA (*n* = 16)	VitA (*n* = 15)	IFA (*n* = 12)	IFA + vitA (*n* = 12)	Within Group Over Time	Between Group	SCI	Group × SCI
**Hb(g/L)**									<0.001	0.181	0.325	0.027
Baseline	9.0 ± 1.3	8.9 ± 1.1	8.9 ± 1.1	8.8 ± 1.2	8.6 ± 1.3	9.1 ± 1.4	8.3 ± 1.1	8.9 ± 1.2				
Midline	9.9 ± 1.1	9.8 ± 0.9	9.8 ± 1.0	9.9 ± 1.0	9.7 ± 1.0	10.4 ± 1.1	9.7 ± 1.6	9.8 ± 1.2				
Endline	11 ± 0.8	10.9 ± 0.9	11.2 ± 0.9	11.1 ± 0.9	10.7 ± 0.8 ^b^	11.2 ± 1.1 ^b^	10.4 ± 1 ^a^	11.3 ± 0.9 ^b^				
**SF (µg/L) ^3^**									0.178	0.154	<0.002	0.444
Baseline	43.7 ± 46.2	43.4 ± 33.3	40.7 ± 28.0	36.9 ± 28.9	54.5 ± 46.7	79.8 ± 59.9	67.7 ± 51.9	65.9 ± 52				
Midline	34.2 ± 32.0	39.5 ± 29.6	39.8 ± 22.9	38.1 ± 25.9	46.0 ± 40.1	65.5 ± 38.6	51.4 ± 27.9	56.5 ± 28.4				
Endline	38.2 ± 34.8	44.4 ± 31.3	47.3 ± 29.1	45.4 ± 30.6	50.7 ± 44.6	68.9 ± 42	68.2 ± 48.7	59 ± 42				
**sTfR(mg/L)**									<0.001	0.039	0.129	0.013
Baseline	7.3 ± 5.8	7.2 ± 3.1	7.5 ± 4.5	8.3 ± 5.7	8.8 ± 4.7	6.3 ± 1.6	11.2 ± 9	7.5 ± 4.5				
Midline	7.1 ± 5.7	7.0 ± 3.3	7.0 ± 4.4	7.7 ± 5.0	9.1 ± 6.1 ^b^	5.9 ± 1.4 ^a^	11.0 ± 8.3 ^b^	6.6 ± 2.9 ^a^				
Endline	7.2 ± 5.5	6.9 ± 3.2	6.8 ± 4.2	6.9 ± 3.8	9.0 ± 4.9 ^b^	5.7 ± 1.3 ^a^	10.5 ± 8.2 ^b^	6.1 ± 2.0 ^a^				
**Body Iron Store (mg/kg)**									0.003	0.110	0.054	0.128
Baseline	4.1 ± 3.5	4.1 ± 4.3	3.9 ± 3.7	2.9 ± 5.2	3.8 ± 5.3	6.9 ± 2.8	4.4 ± 5.1	5.1 ± 5.5				
Midline	3.5 ± 3.3	3.9 ± 4.0	4.5 ± 2.9	3.9 ± 3.7	3.6 ± 3.9 ^a^	6.8 ± 2.3 ^b^	4.0 ± 4.1 ^a^	6.0 ± 3 ^b^				
Endline	3.9 ± 3.7	4.4 ± 4.2	5.1 ± 3.1	4.6 ± 4.0	3.3 ± 5.4	6.8 ± 3.1	4.8 ± 4.6	5.9 ± 3.7				
**Serum Retinol (µmol/L) ^3^**									0.052	0.329	0.338	0.80
Baseline	1.23 ± 0.36	1.22 ± 0.32	1.25 ± 0.31	1.19 ± 0.31	1.08 ± 0.22	1.1 ± 0.29	1.14 ± 0.27	1.32 ± 0.33				
Midline	1.12 ± 0.31	1.19 ± 0.35	1.18 ± 0.29	1.18 ± 0.32	1.08 ± 0.3	1.17 ± 0.34	1.28 ± 0.31	1.15 ± 0.27				
Endline	1.11 ± 0.32	1.23 ± 0.35	1.18 ± 0.34	1.19 ± 0.34	1.03 ± 0.16	1.13 ± 0.23	1.1 ± 0.32	1.16 ± 0.28				

^1^ Values were presented as mean ± SD. ^ab^ Values with different superscript letters are significantly different, *p* < 0.05, FA: Folate group, VitA: Vitamin A and folate, IFA: Iron and folate group, IFA + VitA: Iron folate and vitamin A group; ^2^ Repeated measures ANOVA with the test for 2-factor interaction, significant at *p* < 005; ^3^ Transformed data for SF, sTfR, and Serum Retinol were used for analyses.
